# Evaluating the expression level of miR-9-5p and miR-192-5p in gastrointestinal cancer: introducing novel screening biomarkers for patients

**DOI:** 10.1186/s13104-020-05071-9

**Published:** 2020-04-19

**Authors:** Shaian Tavakolian, Hossein Goudarzi, Ebrahim Faghihloo

**Affiliations:** Department of Microbiology, School of Medicine, Shahid Beheshti University of Medical Sciences, Tehran, 37517 Iran

**Keywords:** miR-9-5p, miR-192-5p, Gastric cancer, Colon cancer, Real-time PCR

## Abstract

**Objective:**

It has been indicated that there is a tight association between cancer and different factors, such as environment and genetics, including aberrantly expressed microRNAs. The crucial role of microRNAs in the regulation of diverse signaling pathways in gastrointestinal cancer has been established in several studies. In this study, we aimed to evaluate the expression of microRNA-9 and -192 in colon and gastric cancers. After extracting the RNA from tissues and serum samples of patients, suffering from colon and gastric cancer, cDNA was synthesized. Then by performing quantitative real-time PCR, we evaluated the expression level of miR-9-5p and miR-192-5p in collected samples.

**Results:**

Unlike to colon cancer in which the expression level of miR-9-5p remained unchanged, the relative expression of this miRNA decreased remarkably in gastric cancer (with *P* value < 0.05), in comparison with normal adjacent tissues. In agreement with this finding, we also found that the expression level of miR-192-5p was decreased in gastric cancer tissues, compared to normal gastric tissue. Given the reduction in the expression level of miR-9-5p and miR-192-5p in gastric cancer, it could be postulated to consider these miRNAs as promising diagnostic biomarkers.

## Introduction

The critical role of environmental factors, including pollution, radiation, high fat in diet and infectious diseases, in the development and the progression of the human cancer is well-established in numerous studies, however, the significant effect of genetic factors on the initiation of different malignancies should not be underestimated [1–6]. Once the discovery of microRNAs, a small noncoding RNAs that act as endogenous silencers of numerous target genes [7], their remarkable role in the regulation of wide range of signaling pathways has been identified [8]. Although some microRNAs serve as oncogenes and activate cancer-related signaling pathways, the others could participate as tumor suppressors, affecting the expression of various biomolecules, such as neuropilin‑1, metalloproteinase 14 (MMP-14), Snail and so forth that are important for the formation of the malignancies [9–12]. Moreover, the unique profile of microRNA expression in human cancer cells and their tissue-specific manner made these small RNAs a potent biomolecules for both cancer diagnosis and screening [13]. The importance of miRNAs in the current cancer studies is not only restricted to cancer screening and lately intense interest has been devoted into miRNAs targeting as a novel strategies in cancer treatment [14].

In the longitude list of microRNAs, the expression level of both miR-9-5p and miR-192-5p has been shown to have an association with cancers. It has been indicated that miR-9 could regulate the expression of E-cadherin, a prominent molecule responsible for cell adhesion, in breast cancer cells [15, 16]. Moreover, another study showed that the over-expression of miR-192 in liver cancer cells not only increased the expression level of E-cadherin, but also modulated the angiogenesis process [17]. Garofalo et al. proposed that microRNAs participate in the pathogenesis of gastrointestinal cancer through regulating fundamental biological processes, such as proliferation and apoptosis [18]. Among different gastrointestinal cancers, gastric and colon cancer are the most prevalent ones by accounting for the 9% and 10% of invasive cancer, respectively [19]. In a study conducted by Bandres et al. it has been indicated that down-regulation of miR-451 was associated with worse prognosis in both gastric and colorectal cancer [20]. In another study, it has been proposed that while the expression level of miR-17-3p and miR-92 was raised in the patients with colorectal cancer, the plasma levels of these microRNAs significantly declined after surgery; introducing aforementioned microRNAs as a minimally-invasive molecular markers for colorectal screening [21]. Although multiple lines of evidence emphasized the significant role of miRNAs in different kind of cancers, as far as we are aware, the relationship between the expression of miR-9-5p, miR-192-5p (mature miRNA) and gastrointestinal cancer remains open to debate. In this study, we aimed to evaluate the expression of aforementioned miRNAs in samples collected from colon and gastric cancer patients.

## Main text

### Ethical statement

Approval to conduct this study was obtained from the Shahid Beheshti University of Medical Sciences” (IR.SBMU.MSP.REC.1397.552, Grant No. 14315).

### Sample collection

In this case–control study, we collected serum and fresh biopsy tissues of patients suffering from gastric and colon cancer (27 gastric and 21 colon cancer tissues, serum and their adjacent normal tissues) who were hospitalized at Emam hossein hospital in Tehran between 2017 and 2018. All samples were stored in RNA later (Qiagen GmbH, Hilden, Germany) in − 20 °C. Furthermore, patient’s serum was collected and stored in − 70 °C until use. All personal information, including all patient’s clinic pathological data, were summarized in Table 1. The stage of the cancers was examined by expert pathologists. The samples of patients who had a history of chemotherapy were excluded from the study.

**Table 1:** The pathological information of gastric and colon samples

	Gastric cancer tissue	Colon cancer tissue	Normal adjacent gastric tissue	Normal adjacent colon tissue
Female	10	13	8	10
Male	15	5	17	8
Mean age	68.3	62.4	65.4	63
Well differentiated adenocarcinoma	11	10		
Moderate and poor differentiate adenocarcinoma	14	8		
*H. Pylori* positive	5	Unknown	4	Unknown

### RNA extraction

To extract RNA, all tissues samples have been dissolved in RNX-plus solution by glass mortar, and proteins were removed from tissues and serum by RNX-plus solution (Cinnagen, Tehran, Iran) and chloroform. (After adding RNX-plus solution and chloroform and centrifuging all samples, RNA was available in upper solution.) Thereafter, RNA was precipitated with isopropanol, and washed with 70% ethanol. Finally, RNAs were dissolved in 50 μl of sterile water. The extracted RNAs were quantified by a Nanodrop spectrophotometry (Eppendorf, Humburg, Germany) at the wavelength of 260 nm. We used 1% agarose gel electrophoresis to confirm the purity of RNA.

#### Stem-loop real-time polymerase chain reaction (RT-PCR) for miRNA-9, -192 in tissue and serum samples

A same panel of two miRNAs (miR-9, miR-192) was employed to RT-PCR analysis in the tissue and serum samples along with their adjacent normal tissues. An equal volume of the eluted RNA and reverse transcriptase BioFACT were combined. Actually, to synthesize cDNA, we combined 8.5 μl of reverse transcriptase BioFACT (Daejeon, South Korea), 10 μl of RNA, 0.5 μl primer (10 pmol) of RTmicroRNA-9 (5′-GTCGTATCCAGTGCAGGGTCCGAGGTATTCGCACTGGATACGACTCATAC-3′), 0.5 μl primer (10 pmol) of RTmicroRNA-192 (5′-GTCGTATCCAGTGCAGGGTCCGAGGTATTCGCACTGGATACGACGGCTGT-3′) and 0.5 μl (10 pmol) of the u6 reverse primer (5′-ATATGGAACGCTTCACGAATTTGC-3′). (choosing a stem-loop primer). All samples were incubated for 5 min at 95 °C and 40 min at 50 °C according to the manufacturer’s recommendations. In order to make suitable concentration, we diluted cDNAs two times with sterile water, and the final volume of sterile water and cDNA sample was 40 μl.

### Quantitative RT-PCR (qRT-PCR)

Quantitative real-time PCR evaluated the accurate microRNA expression. Quantitative real-time PCR was performed by Rotor-gene 6000 (Corbett life sciences, Sydney, Australia) in 36-well Gene Discs, using a final volume of 20 μl. We have combined 10 μl of BIOFACT™ 2X real-time PCR master mix (for SYBR Green I; BIOFACT, South Korea), 1 μl (10 pmol) of forward primer, 1 μl of (10 pmol) reverse primer, 2 μl of 1/2 diluted cDNA and 6 μl of sterile water to evaluate the expression of miR-9-5p and miR-192-5p. U6 was employed to normalize the RNA input (normalization procedure was the same for both serum samples and tissues). To confirm our results, all experiments have been carried out in triplicate, simultaneously. (the same protocol was used to determine microRNAs expression levels both in serum and tissues). The list of primers used in this study are summarized in Table 2. Thermal cycling conditions was 95 °C for 10 min followed by 40 cycles of 95 °C for 20 s, 55 °C for 20 s, and 72 °C for 20 s. The melting curve analysis was performed with ramping 65–95 °C (raising by 0.5 °C each step). The values for the relative quantification were calculated based on 2^−ΔΔct^ expression formula.

**Table 2:** Nucleotide sequences of primers used for real-time RT-PCR

Gene	Forward primer (5′–3′)	Reverse primer (5′–3′)
U6	GAGAAGATTAGCATGGCCCCT	ATATGGAACGCTTCACGAATTTGC
miR-9	CTTTGGTTATCTAGCTGTATGAGTCGT	ATCCAGTGCAGGGTCCGA
miR-192	CTGACCTATGAATTGACAGCCGT	ATCCAGTGCAGGGTCCGA

### Statistical analysis

To analysis the results of miRNAs expression, we used Graph-PadPrism software (version 5.1). Experimental data was expressed by mean ± standard deviation of three independent assays. The unpaired, two-tailed Student’s *t* test was used to analyze the statistical differences between groups using Graph-Pad Prism software. P < 0.05 was considered to indicate a statistically significant difference.

### Results

#### Sample

As we could not extract the RNA of all tissues, some of the samples were excluded from further analyses. To evaluate the expression of miR-9-5p and miR-192-5p in gastric and colon cancer, we included 25 samples of gastric cancer (15 male, 10 female with the mean age of 68.3), adjacent normal gastric tissues (17 male, 8 female with mean age of 65.4), 18 colon cancer samples (5 male, 13 female; with the mean age of 62.4) and their adjacent normal ones (8 male, 10 female with mean age of 63). Moreover, the serum samples of each patient were collected. (27 gastric and 21 colon cancer) Of note, based on the clinical status, among gastric cancer tissues, 5 were considered as poorly differentiated, 9 as moderate differentiation and 11 of samples were well-differentiated adenocarcinoma. Likewise, 3 of the colon cancer samples were poorly differentiated, 5 displayed moderate differentiation and 10 were well-differentiated adenocarcinoma.

#### The expression level of miR-9-5p decreased in gastric cancer

**Fig. 1:**
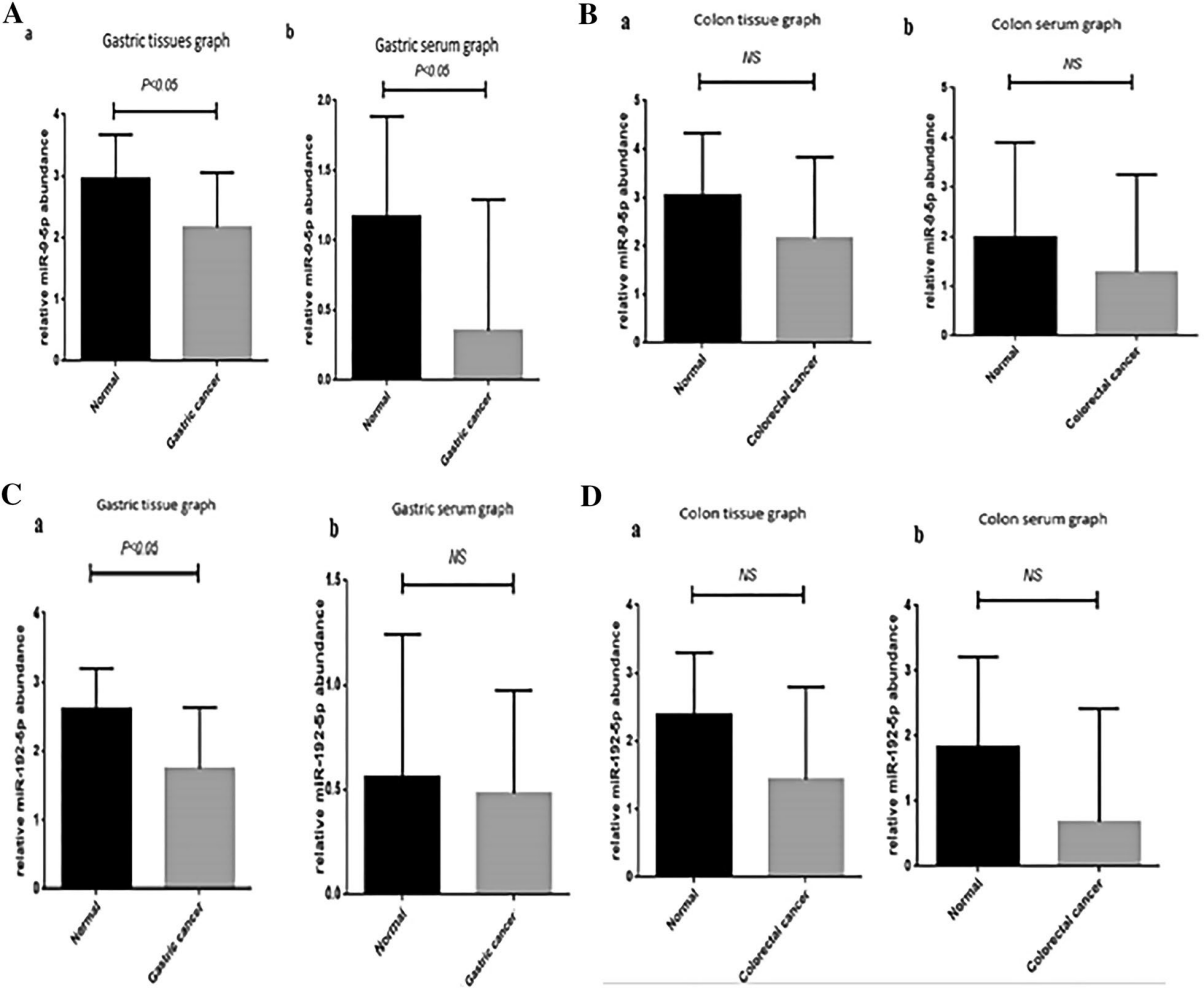
**A** a) The expression level of miR-9-5p significantly decreased in the tissues of gastric cancer (*P* ≤ 0.05) in comparison with the 25 normal tissues. b) There was also a reduction in the expression level of miR-9-5p in the serum of gastric cancer patients (*P* ≤ 0.05). Values are given as mean ± S.D. of three independent experiments. **B** a) Analyzing the expression level of miR-9-5p in colon cancer shown there was no noticeable difference between the expression level of miR-9-5p in the colon tissues samples b). Furthermore, our serum samples demonstrated that the expression level of this miRNA was remained unchanged. **C** a) The expression level of miR-192-5p indicated decrease in the gastric cancer tissues (*P* ≤ 0.05), b) but reduction in serum samples of patients, suffering from gastric cancer was not statistically significant. **D** a, b) The analysis of miR-192-5p expression shown that the level of miR-192-5p was not significantly reduced, neither in colon cancer tissues nor serum samples when compared with normal counterparts

To evaluate the expression level of miR-9-5p in both gastric and colon cancer, we performed real-time PCR analysis. As depicted in Fig. 1, our data showed that the expression level of miR-9-5p significantly decreased in gastric cancer tissues in comparison with the normal counterparts (*P* ≤ 0.05). This finding was further confirmed by the examination of the amount of miR-9-5p in the patient’s serum, which showed a remarkable reduction in miR-9-5p level (*P* ≤ 0.05) (Fig. 1). Unlike the gastric cancer, the expression level of miR-9-5p remained unchanged in both tissue and serum of colon cancer (Fig. 1).

#### miR-192-5p expression was reduced in gastric cancer tissues

Examination of miR-192-5p in gastric cancer showed that the expression level of this miRNA statistically decreased in cancerous tissues; however, we could not find any alteration in the expression level of miR-192-5p in the serum samples (Fig. 1). Moreover, we found that there was no significant difference between miR-192-5p expression levels in normal and colorectal cancer tissue (Fig. 1).

### Discussion

Through binding to the specific sites, especially 3′UTR of mRNAs, microRNAs could robustly enhance or silence the expression level of wide range of genes, which are involved in different signaling pathways. This unique interactions categorized miRNAs into two groups, oncomiRs or tumor suppressors [22, 23]. Given to the well-established role of microRNAs in the pathogenesis of human cancers, these small non-coding RNAs are now considered as a promising biomarkers [24].

Previous studies has demonstrated that there is an association between the expression level of miR-9 and gastrointestinal cancers [20, 21, 25]. In the current study, we found that the expression level of miR-9-5p is significantly reduced both in the tissue and the serum of gastric cancer patients. This finding was in agreement with the recent studies suggesting that the expression level of miR-9 is downregulated in gastric cancer [26–28]. It seems, the evaluation of miR-9-5p in serum of patients, suffering from gastric cancer, can be considered as a biomarker. This result was similar to some studies which monitored different microRNAs in serum samples [29, 30]. The stimulatory of miR-9 in cancer progression is not only restricted to gastric cancer, since other studies also suggested that the expression of this miRNA could be altered in other human malignancies, including squamous cell carcinoma [31], breast cancer [32] and non-small-cell lung cancer [33]. Wan-Cheng Xiong et al. has demonstrated that the up-regulation of miR-9 in colon cancer cells is coupled with the suppression of tumor growth and cancer progression [23]. Moreover, in another study, it has been suggested that miR-9 could inhibit cell migration in colon cancer cells through down-regulation of TM4SF [34]. Of great interest, although the correlation between down-regulation of miR-9 and colon cancer has been examined in different studies, in many cases there are conflicting results. While a previous report indicated that miR-9 is down-regulated in colon cancer cells, other studies reported that miR-9 is over-expressed in colorectal cancer tissues [35, 36]. Given these, it was tempting to evaluate the expression level of miR-9 in colon cancer samples. Of particular interest, the results of our study delineated that there is no correlation between the expression level of miR-9 in, either in the tissue or in the serum of colon cancer.

In this study, we also evaluated the expression level of miR-192-5p. We found that the expression level of this miRNA remarkably decreased in gastric cancer tissues, proposing it as a promising screening biomarker. However, in colon cancer, the expression level of this miRNA remained unchanged. The previous results, showing a correlation between the expression of miR-192-5p and different cancers, displayed a controversially results in gastrointestinal cases. Chiang et al. has indicated that the expression of miR-192 decreased in colon cancer tissues [37]. Moreover, analyzing the amount of miR-192 in rat colon adenocarcinoma delineated the same results [38]. Yeunpo Chiang has also claimed a decrease in the expression level of miR-192 in gastric cell lines (MGC-803 cells, BGC-823 cells); however, they could not find any changes in the expression level of this miRNA in gastric cancer tissues [39]. Unlike these investigations, other studies has declared that the amount of miR-192 is increased in the plasma of gastric cancer patients [40, 41], human hepatocellular carcinoma [42] and non-small cell lung cancer [43]. The resulting data suggested that the expression of both miR-9-5p and miR-192-5p is down-regulated in gastric cancer, suggesting that these microRNAs could serve as a promising screening biomarker in gastric cancer, but not in colon cancer.

## Limitation

The low number of sample was considered as the limitation of this study.

## Data Availability

All data generated or analyzed during this study are included in this published article and its additional files.

## Abbreviations

miR-9-5p: microRNA-9-5-p
miR-192-5p: microRNA-192-5-p
